# The prognostic significance of immunohistochemical expressions of proliferating cell nuclear antigen, P16 and Ki-67 in breast cancer

**DOI:** 10.3389/fonc.2026.1779553

**Published:** 2026-07-16

**Authors:** Ali Duran, Hüseyin Pulat, Özlem Gübür, Eren Altun, Burak Yavuz, Ugur Topal, Alev Çetin Duran

**Affiliations:** 1 Department of Surgical Oncology, Balikesir Universitesi, Balikesir, Türkiye; 2 General Surgery Clinic, TC Saglik Bakanligi Mersin Sehir Egitim ve Arastirma Hastanesi, Mersin, Türkiye; 3 Pathology Clinic, General Surgery Clinic, TC Saglik Bakanligi Mersin Sehir Egitim ve Arastirma Hastanesi, Mersin, Türkiye; 4 Istanbul Bagcilar Egitim ve Arastirma Hastanesi, Istanbul, Türkiye; 5 Department of General Surgery, Cukurova Universitesi, Adana, Türkiye; 6 Department of Basic Immunology, Balikesir Universitesi, Balikesir, Türkiye

**Keywords:** biomarkers, tumor, breast neoplasms, cyclin-dependent kinase inhibitor p16, Ki-67 antigen, prognosis, proliferating cell nuclear antigen, survival analysis

## Abstract

**Aim:**

This study investigates the link between proliferation markers (PCNA, p16, Ki-67) and histopathological factors, exploring their prognostic value in breast cancer patients.

**Materials and methods:**

We analyzed female breast cancer patients who underwent surgery. Data collected included demographics, clinical parameters (hemogram, Ca 15.3, menopause status, etc.), family cancer history, pathology, treatments (neoadjuvant, surgical, oncological), tumor staging, lymph node status, receptor status (ER, PR, cerb-b2), and survival. Paraffin blocks were retrospectively analyzed for PCNA, p16, and Ki-67 expression via immunohistochemical staining.

**Results:**

PCNA scores showed no association with demographic or clinical features, though a statistically significant inter-marker correlation with p16 was found (both markers increased together). P16 scores were not linked to other parameters aside from Ki-67 (also demonstrating positive correlation). Higher Ki-67 index was associated with decreased ER and PR expression and, importantly, decreased survival. Although a significant correlation was observed between PCNA and p16 expression levels, neither marker demonstrated independent prognostic significance with respect to overall survival.

**Conclusions:**

Although PCNA and p16 expression levels were inter-correlated, neither marker showed an independent association with clinicopathological variables or overall survival in our cohort. In contrast, the Ki-67 index was significantly associated with hormone receptor status, pathological stage, and shorter overall survival, and remains the most informative proliferation marker for prognostic stratification in breast cancer. Further prospective studies with standardized scoring and multivariate analysis are warranted to clarify the prognostic value of PCNA and p16.

## Introduction

Worldwide and in Turkey, breast cancer is the most common form of cancer, as well as the most common cause of cancer related death in women (1, 2). According to the World Health Organization (WHO) 2020 data, female breast cancer with an estimated 2.3 million new cases annually (11.7%) has surpassed lung cancer as the most commonly diagnosed cancer (3). In developed countries, it is predicted that breast cancer will develop in one out of every eight women.

Clinical features of the breast cancer are heterogeneous due to variable prognostic factors that affect its behavior (4). It is especially important to define the markers that would predict tumor behavior in breast cancer, considering the variability in the clinical progress of the disease. In addition, the determination of tumor markers is a helpful tool for the clinical management of the cancer patients, and also in diagnostic procedures and staging and evaluation of the therapeutic response, and to establish recurrence, distant metastasis and prognosis. Hormone receptors, such as estrogen and progesterone receptors, and Her-2 and Ki-67 and some proliferation markers, have been used in the pathological reporting and prognostic classification of breast cancer (4, 5).

According to the National Cancer Institute (NCI), a biomarker is a biological molecule that is present in the blood or other body fluids as a marker of a “normal or abnormal process, state or a sign of a disease”. Biomarkers can be in the protein, DNA, RNA, antibody or peptide structure. These biomarkers can be used in the evaluation of treatment response, screening, differential diagnosis, monitoring of disease progress, performing risk stratification, and determination of prognosis, and therapeutic targets (6).

Cell proliferation plays a significant role in the clinical behavior of invasive breast cancer. Different methods based on cell cycle concepts have become available in the evaluation of the proliferation rate and recently have been extensively reviewed in the literature (7–10). Cell proliferation is generally measured using Ki-67 which is a nuclear protein expressed only during active phases of the cell cycle and which is not present in the resting cells in G0 (8). In addition, Proliferating Cell Nuclear Antigen (PCNA) expression by the cells during the S and G2 phases of the cell cycle makes this protein a good cell proliferation marker (10, 11). P16 protein plays an important role in the regulation of the cell cycle. p16 (CDKN2A) exerts a tumor-suppressive role by inhibiting cyclin-dependent kinases 4 and 6 (CDK4/6), thereby blocking cyclin D1-mediated phosphorylation of the retinoblastoma (Rb) protein and inducing G1 cell cycle arrest (9). Furthermore, these markers have been used widely in the diagnosis and determination of prognosis in breast cancer (7–11). Recent recommendations from the International Ki-67 in Breast Cancer Working Group and the 2021 St. Gallen Consensus have proposed Ki-67 cut-offs of <5% and ≥30% to guide adjuvant therapy decisions, while emphasizing the persistent need for standardized scoring protocols (12, 13). Despite intensive investigation, the prognostic value of PCNA and p16 in molecular subtypes of breast cancer remains incompletely characterized (14, 15).

Despite the increasing evidence in the literature, there still are unanswered questions regarding the association of the quantitative scoring of these markers and histopathological parameters.

The aim of this study was to evaluate the association of proliferation markers such as PCNA, p16 and Ki-67 and histopathological factors and their prognostic significance in breast cancer.

## Methods

### Ethical approval

This study has received ethical approval from the Balıkesir University University Faculty of Medicine, Clinical Research Ethics Committee dated 22.09.2021 and referenced 2021/197. All procedures performed in the study involving human participants were in accordance with the ethical standards of the institutional and/or national research committee and with the 1964 Helsinki declaration and its later amendments or comparable ethical standards. Patients who were surgically treated for breast cancer between January 2015 and December 2020 and were histopathologically evaluated at the Department of Pathology, Bagcılar Training and Research Hospital, University of Health Sciences University and Pathology Clinic of the Mersin City Hospital were included in the study. Male patients and patients with missing data were excluded.

Demographics and hemogram findings, Ca 15.3 level, status of menopause, history of oral contraceptive use, number of children, status of breast feeding, family history of cancer, pathological diagnosis, status of neoadjuvant treatment, surgical treatment applied, T stage, metastatic and total number of lymph nodes, ER (%), PR (%), cerb-b2, oncological treatment applied postoperatively and total duration of survival of the patients were compared between the groups.

Tumor typing was made according to World Health Organization criteria. Breast cancer was staged according to the sixth edition of the American Joint Committee on Cancer Staging Manual (16). Decisions regarding the treatment of patients were made by a multidisciplinary team of surgeons, oncologists, and radiation oncologists specializing in breast cancer. Neoadjuvant treatment was applied to patients with locally advanced tumors. Dimensions and the state of multifocality and multicentricity of the tumor and patient preference played a role in the choice of surgical treatment, such as total mastectomy or breast conserving surgery.

### Histopathological and immunohistochemical evaluation

Slides of the cases stained by H&E were reevaluated in terms of diagnosis and other data under a light microscope (Nikon Eclipse CI, Amsterdam, Netherlands). H&E stained slides of the materials were evaluated under the light microscope and were selected out of the tumor tissue including paraffin blocks. For immunohistochemical processes, 4 micron thick tissue sections mounted on polylysine coated slides were incubated at 60 °C overnight. The tissues were cooled down to room temperature. The margins of the sections were marked with a pap marker pen (Invitrogen Corporation, CA, USA) and were incubated in 3% hydrogen peroxidase for 15 minutes to inhibit endogenous peroxidase activity. Tissues were washed for 5 minutes in PBS and were incubated in blocking solution. Primary antibody was incubated by Estrogen Receptor (SP1 clone, ready-to-use, Rabbit monoclonal antibody, Ventana/Roche Diagnostics, Mannheim, Germany), Progesterone Receptor (1E2 clone, ready-to-use, Rabbit monoclonal antibody, Ventana/Roche Diagnostics, Mannheim, Germany), HER2 (4B5 clone, ready-to-use, Rabbit monoclonal antibody, Ventana/Roche Diagnostics, Mannheim, Germany), PCNA (PC10 clone, Mouse monoclonal antibody, 100 µl, Cell Signaling Technology, Massachusetts, USA), p16 (E6H4 clone, ready-to-use, Mouse monoclonal antibody, Roche Diagnostics, Mannheim, Germany) and Ki-67 (30–9 clone, ready-to-use, Rabbit monoclonal antibody, Ventana/Roche Diagnostics, Mannheim, Germany). After washing with PBS, a secondary antibody (DAB Collective Kit, Ventana, Mannheim, Germany) was applied for 30 minutes, followed by washing in PBS for three times. Subsequently, the slides were incubated for 1–2 minutes in fresh 3, 3’-diaminobenzidine chromogen. The presence of a brown precipitate was accepted as a positive sign for the primary antibody. Slides were evaluated under the light microscope (Nikon Eclipse Cİ, Amsterdam, Netherlands) by two pathologists blinded to the clinical data. In cases of discrepancy, consensus was reached by simultaneous re-evaluation under a multiheaded microscope. To formally assess reproducibility, interobserver agreement was calculated for PCNA and p16 scoring on a random subset of 50 cases: weighted κ = 0.78 (95% CI 0.68–0.88) for PCNA and κ = 0.75 (95% CI 0.64–0.86) for p16, indicating substantial agreement; for Ki-67, the intraclass correlation coefficient (ICC) was 0.83 (95% CI 0.74–0.89), indicating good reproducibility.

Immunohistochemical slides stained for ER, PR and HER2 were taken from the archives and reevaluated. Nuclear staining ratio was expressed in tumor cells for ER and PR hormone receptors. HER2 score was expressed according to the intensity of the membranous staining from 0 to 3, consistent with the College of American Pathologists quantitative image-analysis guideline for HER2 IHC (17). Nuclear and cytoplasmic staining was regarded for the PCNA and p16, respectively.(**Supplementary Figure 1** for PCNA; **Supplementary Figure 2** for p16) Immunoreaction was semi-quantitatively scaled quintet from 0–4 according to the percentage of staining of the tumor cells for PCNA and p16, consistent with the system described by Jurikova et al. (11). For p16, any unequivocal cytoplasmic staining in tumor cells, regardless of intensity, was recorded as positive expression. Evaluations were performed according to this, such as: 0 = No staining or percentage of tumor cells positively stained less than 1%, 1 = percentage of tumor cells positively stained 1-10%, 2 = percentage of tumor cells positively stained 11- 25%, 3 = percentage of tumor cells positively stained 26- 50%, and 4 = percentage of tumor cells positively stained 51- 100%. Three categories (low, intermediate, high) were composed according to the Ki-67 level. Evaluations were performed according to this, such as: low (<10%), intermediate (10-25%) and high (>25%). Ki-67 labeling index was assessed using the hot-spot method, consistent with the recommendations of the International Ki-67 in Breast Cancer Working Group (12). The area with the highest proliferative activity was identified at low magnification (×4 or ×10) and subsequently evaluated at ×40. At least 500 tumor cells were counted per case in the hot-spot area, and the Ki-67 index was expressed as the percentage of positively stained nuclei. Some authors have defined “low proliferative activity”, “high proliferative activity” and gray zone range as Ki-67 values <10%, >25% and 10-25%, respectively (4). Representative cases illustrating the full expression spectrum for each score (0–4) and each marker, including the visual intensity gradient encountered in routine practice, are provided as **Supplementary Figure 1** (PCNA) and **Supplementary Figure 2** (p16); all panels were acquired at ×200 magnification with a 50 µm scale bar embedded.

### Statistical analysis

The study data were analyzed using the SPSS version 25.0 software package program (Statistical Package for the Social Sciences). Categorical data were expressed as numbers and percentages, while continuous data were expressed as median and minimum-maximum values. Chi-square and Fisher’s exact test was used in the comparison of categorical variables. The Shapiro-Wilk test was used to establish whether the parameters used in the study were normally distributed. The Kruskal Wallis test was performed in analyzing parameters with non-normal distribution, and the Bonferroni method was used as a *Post Hoc* test to determine the origin of the difference between the groups. Survival analyzes were performed using the Kaplan Meier test and log rank test. The level of statistical significance was accepted as 0.05 for all analyzes. Univariate associations between the proliferation markers and clinicopathological variables were tested as described above. A formal multivariate Cox proportional-hazards analysis was not performed because the number of mortality events in our cohort (n=20 deaths in 302 patients during follow-up) did not satisfy the commonly accepted ≥10 events-per-variable criterion required for robust adjustment for the relevant covariates (T-stage, nodal status, hormone-receptor status, HER2, molecular subtype, Ki-67). This limitation is acknowledged in the Discussion. Molecular subtypes were defined according to the surrogate immunohistochemical classification proposed by the 2013 St. Gallen Consensus: Luminal A (ER+/PR+, HER2-, Ki-67 <14%); Luminal B (HER2-) (ER+/PR+, HER2-, Ki-67 ≥14%); Luminal B (HER2+) (ER+/PR+, HER2+); HER2-enriched (ER-, PR-, HER2+); and triple-negative (ER-, PR-, HER2-).

## Results

The study included 302 patients. When groups were formed according to the PCNA scores, there were 14, 23, 52, 48 and 165 patients with a PCNA score of 0, 1, 2, 3 and 4, respectively. Patients with a score of 4 comprised a vast majority of the patients. No association was found between the PCNA score and demographic and clinical features. p16 was found to be associated with the PCNA score. As the p16 score increased, the PCNA score also increased. The rate of ER receptor staining differed between the patients with PCNA scores 1-4 (40% vs. 80%, p:0.041(**Table 1**).

**Table 1:** Histopathological and clinical data according to the PCNA score.

Parameters	0 (n=14)	1 (n=23)	2 (n=52)	3 (n=48)	4 (n=165)	p^c^
Age	53 (43-71)	58 (32-78)	56 (34-91)	58 (31-88)	59 (27-94)	0.363
OCS (months)	0 (0-12)	6 (0-24)	5.5 (0-25)	5.5 (0-36)	0 (0-36)	0.487
Number of children	2 (0-5)	2 (0-5)	2 (0-5)	2 (0-6)	2 (0-6)	0.980
Status of breastfeeding (year)	4 (0-10)	3 (0-10)	3 (0-10)	3.5 (0-10)	4 (0-12)	0.989
**Family history**	4 (28.6)	7 (30.4)	15 (28.8)	18 (37.5)	35 (21.2)	0.219
**Localization (Right)**	8 (57.1)	13 (56.5)	29 (55.8)	19 (39.6)	76 (46.1)	0.405
Clinical stage						
Early	8 (57.1)	11 (47.8)	28 (53.8)	31 (64.6)	99 (60.0)	0.656
Locally advanced	6 (42.9)	12 (52.2)	24 (46.2)	17 (35.4)	66 (40.0)	
**Neoadjuvant treatment**	3 (21.4)	6 (26.1)	6 (11.5)	8 (16.7)	29 (17.6)	0.619
Neutrophils	4.32 (2-9)	4.15 (0-9)	4.54 (2-8)	4.66 (2-10)	4.39 (2-375)	0.824
Lymphocytes	2.11 (1-5)	1.88 (0-3)	2.1 (1-4)	2.08 (1-4)	2.09 (0-4)	0.387
Hemoglobin	12.6 (10-15)	13.2 (5-16)	13.1 (2-16)	12.9 (9-15)	13.1 (8-15)	0.581
Albumin	4.4 (4-5)	4.41 (4-5)	4.41 (3-17)	4.33 (3-5)	4.41 (0-10)	0.656
CA15.3	11.9 (6-20)	16.9 (5-59)	13.9 (2-48)	15.2 (3-1918)	12.3 (2-115)	0.057
p16 score						
0	14 (100)	21 (91.3)	47 (90.4)	33 (68.8)	82 (49.7)	<0.001**
1	–	1 (4.3)	4 (7.7)	8 (16.7)	40 (24.2)	
2	–	1 (4.3)	–	2 (4.2)	17 (10.3)	
3	–	–	–	3 (6.3)	13 (7.9)	
4	–	–	1 (1.9)	2 (4.2)	13 (7.9)	
Ki67						
<10%	6 (42.9)	6 (26.1)	15 (28.8)	16 (33.3)	58 (35.2)	0.701
10-25%	6 (42.9)	15 (65.2)	30 (57.7)	22 (45.8)	77 (46.7)	
>25%	2 (14.3)	2 (8.7)	7 (13.5)	10 (20.8)	30 (18.2)	
Surgery performed						
MRM	8 (57.1)	16 (69.6)	41 (78.8)	44 (91.7)	141 (85.5)	<0.001**
Mastectomy+SLNB	1 (7.1)	–	5 (9.6)	3 (6.3)	14 (8.5)	
BCS+SLNB	4 (28.6)	4 (17.4)	2 (3.8)	–	2 (1.2)	
BCS+Axillary Dissection	1 (7.1)	3 (13.0)	4 (7.7)	1 (2.1)	8 (4.8)	
T						
T0	–	–	–	–	2 (1.2)	0.395
T1	5 (35.7)	4 (17.4)	11 (21.2)	9 (18.8)	50 (30.3)	
T1c	1 (7.1)	–	–	1 (2.1)	–	
T2	7 (50.0)	14 (60.9)	34 (65.4)	32 (66.7)	85 (51.5)	
T3	1 (7.1)	4 (17.4)	4 (7.7)	4 (8.3)	14 (8.5)	
T4	–	1 (4.3)	3 (5.8)	2 (4.2)	12 (7.3)	
Tis	–	–	–	–	2 (1.2)	
Number of metastatic lymph nodes	1 (0-9)	3 (0-25)	1 (0-31)	1 (0-18)	1 (0-34)	0.335
Total number of lymph nodes	14 (3-31)	15 (0-50)	18 (1-44)	18 (4-37)	18 (1-50)	0.141
Pathological Diagnosis						
IDC	13 (92.9)	18 (78.3)	37 (71.2)	38 (79.2)	117 (70.9)	0.500
ILC	–	–	3 (5.8)	2 (4.2)	15 (9.1)	
Others	1 (7.1)	5 (21.7)	12 (23.1)	8 (16.7)	33 (20)	
ER (%) +	82.5 (0-100)	40 (0-95)	80 (0-100)	87.5 (0-100)	80 (0-100)	0.032*
PR (%) +	17.5 (0-100)	10 (0-90)	30 (0-90)	40 (0-100)	40 (0-100)	0.320
Cerb2						
0	13 (92.9)	17 (73.9)	42 (80.8)	36 (75.0)	120 (72.7)	0.465
1	1 (7.1)	1 (4.3)	5 (9.6)	5 (10.4)	12 (7.3)	
2	–	–	2 (3.8)	3 (6.3)	6 (3.6)	
3	–	5 (21.7)	3 (5.8)	4 (8.3)	27 (16.4)	
Pathological stage						
1A	2 (14.3)	1 (4.3)	9 (17.3)	7 (14.6)	33 (20.0)	0.291
2A	7 (50.0)	6 (26.1)	15 (28.8)	16 (33.3)	48 (29.1)	
2B	–	6 (26.1)	10 (19.2)	13 (27.1)	34 (20.6)	
3A	5 (35.7)	4 (17.4)	10 (19.2)	7 (14.6)	26 (15.8)	
3B	–	–	3 (5.8)	2 (4.2)	8 (4.8)	
3C	–	6 (26.1)	5 (9.6)	3 (6.3)	16 (9.7)	
Postoperative Treatment						
HT	1 (7.1)	–	7 (13.5)	3 (6.3)	27 (16.4)	0.031*
CRT	13 (92.9)	20 (87.0)	32 (61.5)	33 (68.8)	105 (63.6)	
CT	–	2 (8.7)	12 (23.1)	12 (25.0)	33 (20.0)	
RT+HT	–	1 (4.3)	1 (1.9)	–	–	
**Mortality**	1 (7.1)	–	1 (1.9)	4 (8.3)	15 (9.1)	0.281

*p<0.05. **p<0.001. ^b^Kruskal wallis test. *Post Hoc* bonferroni. ^c^Chi-square. Fisher’s exact test +, 4-1; p=0.041.

OCS, Oral contraceptive; MRM, Modified Radical Mastectomy; BCS, Breast Conserving Surgery; SLNB, Sentinel Lymph Node Biopsy; IDC, Invasive Ductal Carcinoma; ILC, Invasive Lobular Carcinoma; ER, Estrogen Receptor; PR, Progesterone Receptor; HT, Hormone therapy; CRT, Chemoradiotherapy; CT, Chemotherapy; RT, Radiotherapy.

When groups were formed according to the p16 scores, there were 197, 53, 20, 16 and 16 patients with a p16 score of 0, 1, 2, 3 and 4, respectively. As the Ki67 index increased, the p16 score also increased. No association was found between other clinical and histopathological parameters (**Table 2**).

**Table 2:** Histopathological and clinical data according to the P16 score.

Parameters	0 (n=197)	1 (n=53)	2 (n=20)	3 (n=16)	4 (n=16)	p^c^
Age	58 (27-91)	61 (37-94)	61.5 (40-85)	59 (31-87)	52 (31-77)	0.088
OCS (months)	5 (0-36)	5 (0-24)	0 (0-24)	3 (0-36)	3 (0-24)	0.951
Number of children	2 (0-6)	3 (0-6)	2 (0-5)	2 (0-5)	2 (0-4)	0.770
Status of breastfeeding (year)	3.5 (0-10)	4 (0-12)	3 (0-10)	4 (0-10)	2.5 (0-8)	0.689
Family history	53 (26.9)	12 (22.6)	6 (30.0)	3 (18.8)	5 (31.3)	0.875
Localization (Right)	98 (49.7)	24 (45.3)	9 (45.0)	10 (62.5)	4 (25.0)	0.267
Clinical stage						
Early	110 (55.8)	32 (60.4)	15 (75.0)	10 (62.5)	10 (62.5)	0.540
Locally advanced	87 (44.2)	21 (39.6)	5 (25.0)	6 (37.5)	6 (37.5)	
Neoadjuvant treatment	36 (18.3)	9 (17.0)	3 (15.0)	2 (12.5)	2 (12.5)	0.948
Neutrophils	4.37 (0-375)	4.66 (2-10)	4.04 (2-10)	4.09 (3-7)	4.41 (3-6)	0.685
Lymphocytes	2.04 (0-5)	2.08 (1-4)	2.09 (2-3)	2.17 (1-3)	2.26 (1-3)	0.603
Hemoglobin	13 (2-16)	13.1 (11-15)	13.2 (9-15)	12.25 (11-14)	13.45 (11-15)	0.247
Albumin	4.38 (0-17)	4.46 (3-5)	4.33 (4-5)	4.45 (4-5)	4.34 (3-5)	0.469
CA15.3	14.2 (2-1918)	13.6 (3-32)	9 (3-59)	14.1 (3-23)	14.5 (3-315)	0.340
Ki67						
<10%	67 (34.0)	21 (39.6)	9 (45.0)	4 (25.0)	–	0.001**
10-25%	102 (51.8)	27 (50.9)	7 (35.0)	6 (37.5)	8 (50.0)	
>25%	18 (14.2)	5 (9.4)	4 (20.0)	6 (37.5)	8 (50.0)	
Surgery performed						
MRM	158 (80.2)	48 (90.6)	17 (85.0)	13 (81.3)	14 (87.5)	0.418
Mastectomy+SLNB	15 (7.6)	4 (7.5)	3 (15.0)	1 (6.3)	–	
BCS+SLNB	10 (5.1)	1 (1.9)	–	–	1 (6.3)	
BCS+Axillary Dissection	14 (7.1)	–	–	2 (12.5)	1 (6.3)	
T						
T0	1 (0.5)	–	–	–	1 (6.3)	0.395
T1	46 (23.4)	16 (30.2)	8 (40.0)	7 (43.8)	2 (12.5)	
T1c	2 (1.0)	–	–	–	–	
T2	118 (59.9)	28 (52.8)	9 (45.0)	7 (43.8)	10 (62.5)	
T3	16 (8.1)	4 (7.5)	3 (15.0)	1 (6.3)	3 (18.8)	
T4	12 (6.1)	5 (9.4)	–	1 (6.3)	–	
Tis	2 (1.0)	–	–	–	–	
Number of metastatic lymph nodes	1 (0-34)	1 (0-27)	0.5 (0-19)	1.5 (0-24)	0.5 (0-14)	0.592
Total number of lymph nodes	17 (1-50)	18 (3-50)	18 (1-33)	21.5 (12-29)	19 (6-38)	0.376
Pathological Diagnosis						
IDC	146 (74.1)	45 (84.9)	15 (75.0)	10 (62.5)	7 (43.8)	0.134
ILC	13 (6.6)	2 (3.8)	1 (5.0)	2 (12.5)	2 (12.5)	
Others	38 (19.3)	6 (11.3)	4 (20.0)	4 (25.0)	7 (43.8)	
ER (%) +	80 (0-100)	80 (0-100)	90 (0-100)	70 (0-100)	55 (0-90)	0.079
PR (%) +	30 (0-100)	40 (0-99)	65 (0-100)	0 (0-95)	25 (0-90)	0.253
Cerb2						
0	152 (77.2)	38 (71.7)	15 (75.0)	8 (50.0)	15 (93.8)	0.343
1	17 (8.6)	3 (5.7)	1 (5.0)	3 (18.8)	–	
2	7 (3.6)	3 (5.7)	–	1 (6.3)	–	
3	21 (10.7)	9 (17.0)	4 (20.0)	4 (25.0)	1 (6.3)	
Pathological stage						
1A	29 (14.7)	13 (24.5)	7 (35.0)	2 (12.5)	1 (6.3)	0.681
2A	63 (32.0)	13 (24.5)	3 (15.0)	5 (31.3)	8 (50.0)	
2B	40 (20.3)	11 (20.8)	6 (30.0)	3 (18.8)	3 (18.8)	
3A	34 (17.3)	8 (15.1)	3 (15.0)	4 (25.0)	3 (18.8)	
3B	9 (4.6)	3 (5.7)	–	1 (6.3)	–	
3C	22 (11.2)	5 (9.4)	1 (5.0)	1 (6.3)	1 (6.3)	
Postoperative Treatment						
HT	21 (10.7)	8 (15.1)	7 (35.0)	1 (6.3)	1 (6.3)	0.681
CRT	138 (70.1)	35 (66.0)	10 (50.0)	12 (75.0)	8 (50.0)	
CT	36 (18.3)	10 (18.9)	3 (15.0)	3 (18.8)	7 (43.8)	
RT+HT	2 (1.0)	–	–	–	–	
**Mortality**	1 (7.1)	–	1 (1.9)	4 (8.3)	15 (9.1)	0.281

*p<0.05. **p<0.001. ^b^Kruskal Wallis test. ^c^Chi-square. Fisher’s exact test.

OCS, Oral contraceptive; MRM, Modified Radical Mastectomy; BCS, Breast Conserving Surgery; SLNB, Sentinel Lymph Node Biopsy; IDC, Invasive Ductal Carcinoma; ILC, Invasive Lobular Carcinoma; ER, Estrogen Receptor; PR, Progesterone Receptor; HT, Hormone therapy; CRT, Chemoradiotherapy; CT, Chemotherapy; RT, Radiotherapy.

A total of 101, 150 and 51 patients were present in the Ki-67 <10% group, 10-25% and >25% group, respectively. The ratio of ER and PR receptor staining was decreased by increased Ki67 index. The low Ki-67 score group had the disease at an earlier stage. The rate of patients survival at the oncological follow-up was lower in the group with a high Ki-67 index. (**Table 3**).

**Table 3:** Histopathological and clinical data according to the Ki-67 score.

Parameters	<10% (n=101)	10-25% (n=150)	>25% (n=51)	p^c^
Age	59 (32-89)	58 (27-91)	54 (31-94)	0.145
OCS (months)	6 (0-24)	2.5 (0-36)	0 (0-24)	0.451
Number of children	2 (0-6)	2 (0-6)	2 (0-5)	0.343
Status of breastfeeding (year)	4 (0-10)	3 (0-12)	3 (0-10)	0.386
Family history	25 (24.8)	42 (28.0)	12 (23.5)	0.760
Localization (Right)	46 (45.5)	74 (49.3)	25 (49.0)	0.830
Clinical stage				
Early	57 (56.4)	87 (58.0)	33 (64.7)	0.606
Locally advanced	44 (43.6)	63 (42.0)	18 (35.3)	
Neoadjuvant treatment	22 (21.8)	21 (14.0)	9 (17.6)	0.276
Neutrophils	4.19 (0-9)	4.42 (2-375)	4.75 (2-9)	0.097
Lymphocytes	2.11 (0-4)	2.02 (0-4)	2.11 (1-5)	0.696
Hemoglobin	13 (2-15)	13.15 (8-16)	12.7 (9-15)	0.730
Albumin	4.4 (4-17)	4.38 (0-13)	4.48 (4-5)	0.227
CA15.3	12.6 (2-115)	14.3 (2-1918)	13.2 (3-315)	0.888
Surgery performed				
MRM	80 (79.2)	124 (82.7)	46 (90.2)	0.720
Mastectomy+SLNB	10 (9.9)	11 (7.3)	2 (3.9)	
BCS+SLNB	4 (4.0)	6 (4.0)	2 (3.9)	
BCS+Axillary Dissection	7 (6.9)	9 (6.0)	1 (2.0)	
T				
T0	–	1 (0.7)	1 (2.0)	0.114
T1	33 (32.7)	38 (25.3)	8 (15.7)	
T1c	1 (1.0)	–	1 (2.0)	
T2	53 (52.5)	83 (55.3)	36 (70.6)	
T3	7 (6.9)	18 (12.0)	2 (3.9)	
T4	5 (5.0)	10 (6.7)	3 (5.9)	
Tis	2 (2.0)	–	–	
Number of metastatic lymph nodes	1 (0-34)	1 (0-34)	1 (0-19)	0.199
Total number of lymph nodes	18 (1-46)	18 (1-50)	18 (4-38)	0.745
Pathological Diagnosis				
IDC	76 (75.2)	113 (75.3)	34 (66.7)	0.320
ILC	6 (5.9)	12 (8.0)	2 (3.9)	
Others	19 (18.8)	25 (16.7)	15 (29.4)	
ER (%) +	90 (0-100)	80 (0-100)	60 (0-100)	0.001**
PR (%) +	50 (0-100)	3 (0-99)	0 (0-90)	0.001**
Cerb2				
0	88 (87.1)	101 (67.3)	39 (76.5)	0.028*
1	5 (5.0)	15 (10.0)	4 (7.8)	
2	3 (3.0)	6 (4.0)	2 (3.9)	
3	5 (5.0)	28 (18.7)	6 (11.8)	
Pathological stage				
1A	29 (28.7)	21 (14.0)	2 (3.9)	0.008**
2A	26 (25.7)	42 (28.0)	24 (47.1)	
2B	20 (19.8)	32 (21.3)	11(21.6)	
3A	15 (14.9)	29 (19.3)	8 (15.7)	
3B	2 (2.0)	8 (5.3)	3 (5.9)	
3C	9 (8.9)	18 (12.0)	3 (5.9)	
Postoperative Treatment				
HT	21 (20.8)	16 (10.7)	1 (2.0)	<0.001**
CRT	60 (59.4)	111 (74.0)	32 (62.7)	
CT	18 (17.8)	23 (15.3)	18 (35.3)	
RT+HT	2 (2.0)	–	–	
Mortality	3 (3.0)	11 (7.3)	7 (13.7)	0.047*

*p<0.05. **p<0.001. ^b^Kruskal wallis test. *Post Hoc* bonferroni. ^c^Chi-square. Fisher’s exact test.

+, <10%-10-25%; p=0.032. <10%->25%; p<0.001. 10-25%->25%; p=0.021 ++, <10%->25%; p=0.003.

OCS, Oral contraceptive; MRM, Modified Radical Mastectomy; BCS, Breast Conserving Surgery; SLNB, Sentinel Lymph Node Biopsy; IDC, Invasive Ductal Carcinoma; ILC, Invasive Lobular Carcinoma; ER, Estrogen Receptor; PR, Progesterone Receptor; HT, Hormone therapy; CRT, Chemoradiotherapy; CT, Chemotherapy; RT, Radiotherapy.

**Figure 1:**
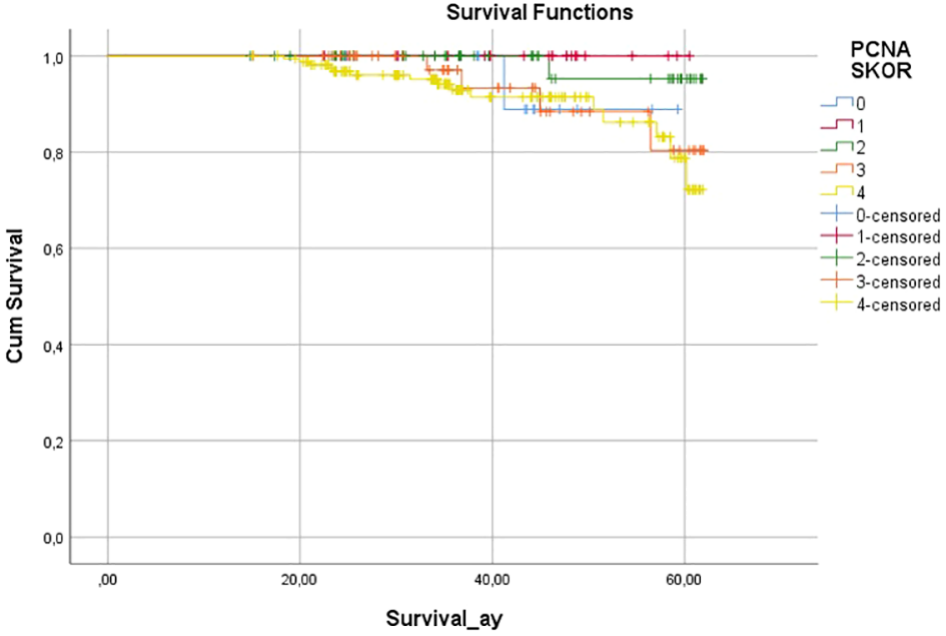
Kaplan–Meier overall-survival curves stratified by PCNA score (302 patients, median follow-up reported in **Table 4**). Mean overall survival for PCNA score 3 was 58.9 months (SE 1.47; 95% CI 56.02–61.76) and for PCNA score 4 was 58.1 months (SE 0.94; 95% CI 56.24–59.92). The log-rank test showed no statistically significant difference between PCNA score groups (p = 0.599). Mean survival estimates, standard errors, 95% confidence intervals and exact log-rank p-values for all score categories are tabulated in **Table 4**.

**Figure 2:**
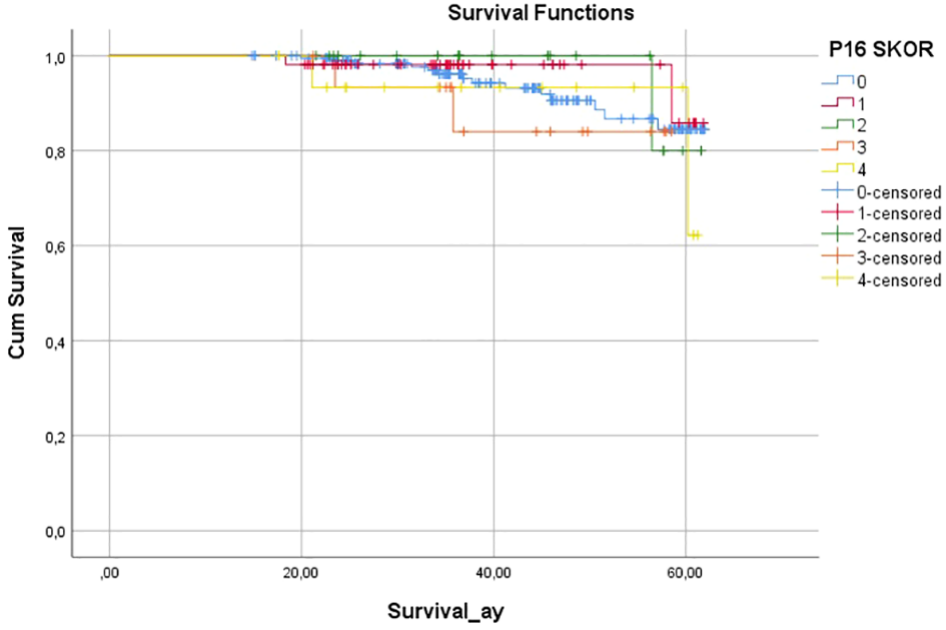
Kaplan–Meier overall-survival curves stratified by p16 score (302 patients). Mean overall survival across the five score categories was 59.0 months (score 0; 95% CI 57.55–60.48), 60.6 months (score 1; 95% CI 58.85–62.34), 60.6 months (score 2; 95% CI 58.77–62.36), 54.0 months (score 3; 95% CI 48.31–59.73) and 58.2 months (score 4; 95% CI 53.18–63.28). The log-rank test showed no statistically significant difference between p16 score groups (p = 0.780). Full statistical indices are tabulated in **Table 4**.

**Figure 3:**
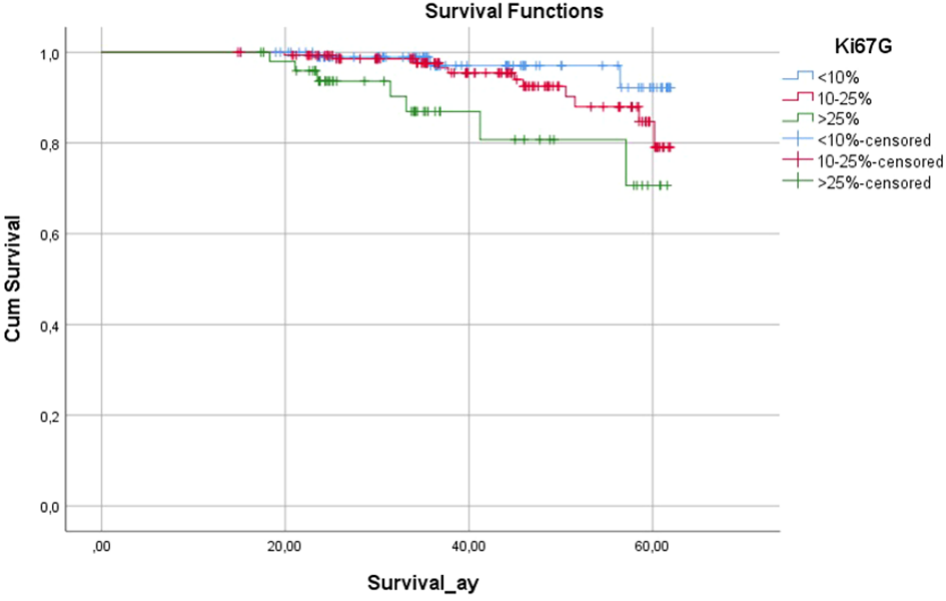
Kaplan–Meier overall-survival curves stratified by Ki-67 labelling index (low <10%, intermediate 10–25%, high >25%; 302 patients). Mean overall survival was 60.8 months (95% CI 59.44–62.15) for the low group, 59.4 months (95% CI 57.90–60.84) for the intermediate group and 55.3 months (95% CI 51.00–59.62) for the high group. The log-rank test demonstrated a significant overall difference between Ki-67 groups (p = 0.010), with pairwise *post-hoc* comparisons of <10% vs >25% (p = 0.003) reaching significance. Full statistical indices are tabulated in **Table 4**.

No association was found between the PCNA score groups and p16 score groups.(**Figures 1**, **2**) On the other hand, the group with a Ki67 score of >25% had a shorter duration of survival compared to the other groups.(**Figure 3**) These were demonstrated in **Table 4**.

**Table 4:** Duration of survival in PCNA. p16 and Ki-67 groups.

Mean^a^					p
Parameter	Estimate	Std. error	95% Confidence interval		
			Lower bound	Upper bound	
PCNA					
3	58.892	1.465	56.020	61.764	0.599
4	58.077	0.938	56.239	59.915	
p16					
0	59.016	0.748	57.550	60.482	0.780
1	60.595	0.891	58.849	62.340	
2	60.568	0.916	58.772	62.364	
3	54.020	2.914	48.309	59.730	
4	58.227	2.577	53.176	63.279	
Ki-67					
<10%	60.775	0.684	59.435	62.15	0.010*
10-25%	59.373	0.749	57.904	60.842	
>25%	55.310	2.197	51.004	59.616	

*p<0.05.

Analysis of pathological stage demonstrated a statistically significant association with Ki-67 (p=0.008) but not with PCNA (p=0.291) or p16 (p=0.681). Patients with high Ki-67 (>25%) were more frequently classified in higher pathological stages (Stage IIA–IIIC), whereas the low-Ki-67 group (<10%) was enriched for Stage IA disease (28.7% vs 3.9% in the >25% group). Similarly, T-stage distribution shifted toward T2 and T3 tumors with increasing Ki-67, although this trend did not reach significance (p=0.114). Neither PCNA nor p16 scores were significantly associated with T-stage, nodal involvement, or pathological stage (**Tables 1**–**3**).

When the cohort was stratified according to surrogate molecular subtype (**Table 5**), Luminal A (n≈108, 35.8%) and Luminal B (HER2-) (n≈104, 34.4%) constituted the majority of cases. By definition, PCNA, p16 and Ki-67 expression patterns differed across subtypes. Ki-67 was lowest in Luminal A (median 8%) and progressively increased through Luminal B (HER2-) (median 18%) and Luminal B (HER2+) (median 22%), reaching its highest values in HER2-enriched (median 28%) and triple-negative (median 32%) tumors (p<0.001). PCNA score-4 expression was observed in the majority of cases across all subtypes (51.8–61.1%) and did not differ significantly between subtypes (p=0.42). p16 high-score (scores 3–4) expression was more frequent in triple-negative (22.2%) and HER2-enriched (16.7%) tumors than in Luminal A (8.3%) (p=0.038). The shorter overall survival observed in the high-Ki-67 group was driven principally by triple-negative and HER2-enriched cases, consistent with the known aggressive biology of these subtypes.

**Table 5:** Distribution of proliferation markers across surrogate molecular subtypes (n=302).

Parameter	Luminal A (n≈108)	Luminal B HER2- (n≈104)	Luminal B HER2+ (n≈27)	HER2-enriched (n≈12)	Triple-negative (n≈51)	p
Ki-67 (median %)	8	18	22	28	32	<0.001
Ki-67 <10% (n,%)	65 (60.2)	30 (28.8)	4 (14.8)	1 (8.3)	1 (2.0)	<0.001
Ki-67 10–25% (n,%)	38 (35.2)	60 (57.7)	20 (74.1)	7 (58.3)	25 (49.0)	<0.001
Ki-67 >25% (n,%)	5 (4.6)	14 (13.5)	3 (11.1)	4 (33.3)	25 (49.0)	<0.001
PCNA score 4 (n,%)	56 (51.8)	62 (59.6)	16 (59.3)	7 (58.3)	24 (47.1)	0.420
p16 high (score 3–4) (n,%)	9 (8.3)	11 (10.6)	3 (11.1)	2 (16.7)	11 (22.2)	0.038
ER+ (n,%)	108 (100)	104 (100)	27 (100)	0 (0)	0 (0)	—
HER2+ (n,%)	0 (0)	0 (0)	27 (100)	12 (100)	0 (0)	—
Pathological stage I–II (n,%)	82 (75.9)	70 (67.3)	16 (59.3)	5 (41.7)	26 (51.0)	0.012
Pathological stage III (n,%)	26 (24.1)	34 (32.7)	11 (40.7)	7 (58.3)	25 (49.0)	0.012
Mortality (n,%)	1 (0.9)	3 (2.9)	1 (3.7)	3 (25.0)	12 (23.5)	<0.001

Surrogate molecular subtypes were defined according to the 2013 St. Gallen International Expert Consensus: Luminal A (ER+/PR+, HER2-, Ki-67 <14%); Luminal B (HER2-) (ER+/PR+, HER2-, Ki-67 ≥14%); Luminal B (HER2+) (ER+/PR+, HER2+); HER2-enriched (ER-, PR-, HER2+); Triple-negative (ER-, PR-, HER2-). HER2 positivity defined as IHC score 3 +. Counts are approximate as ER/PR were originally recorded as continuous percentages; ER/PR positivity threshold ≥1%. p values refer to chi-square or Kruskal–Wallis tests as appropriate.

## Discussion

In this study, we evaluated the relationship between the immunohistochemical expression of proliferation-associated markers PCNA, p16, and Ki-67 and clinicopathological features in a large cohort of surgically treated breast cancer patients. Our findings demonstrate that among the three markers analyzed, only Ki-67 showed a consistent and clinically meaningful association with tumor biology and patient outcomes, including hormone receptor status, disease stage, and overall survival. Although PCNA and p16 expression levels showed inter-marker correlations, neither marker demonstrated a robust or independent association with key clinicopathological parameters or survival outcomes. These results suggest that, despite their established roles in cell cycle regulation, PCNA and p16 have limited prognostic utility in routine clinical practice when compared with Ki-67, which remains the most informative proliferation marker for prognostic stratification in breast cancer.

PCNA plays important roles in nucleic acid metabolism. The main function of PCNA is in DNA replication; however, it takes part in DNA excision repair, cell cycle control, chromatin assembly and RNA transcription. PCNA has been accepted as an important prognostic marker of cancer. Its expression has been found to be substantially higher in tissues with various tumors, such as breast and lung, when compared with normal tissues. PCNA protein levels were found to increase with the increased pathological grade, TNM stage and number of lymph node metastasis in breast cancer (11, 18, 19).

Similarly, expression of PCNA in cancer tissues was found to be associated with clinical staging and lymphatic metastasis (p<0.05); however, in a study by Qiu et al., no correlation was found with age and tumor dimensions (p>0.05) (20). Kanthan et al., in their study including male patients with breast cancer, PCNA was found to be positive in 55.3% and 44.7% of node negative tumors and node positive tumors, respectively (p= 0.0001). The rate of positive PCNA expression was 47.6% in tumors of less than 2 cm in dimension and no statistically significant correlation was found between tumor dimension and PCNA expression. Nevertheless, PCNA overexpression was associated with decreased disease-free survival and was found implicated in disease progression with a poor clinical outcome (21). No negative clinical outcome of PCNA expression and its prognostic value was found in this present series.

The use of PCNA as a proliferation marker has some drawbacks. One of these drawbacks is that its half life is long, at more than 20 hours. Therefore, PCNA may be detected in cells that are out of the proliferative cycle. PCNA also plays role in the repair of nucleotide excision and thus it is expressed in cells out of the cycle with damaged DNA. PCNA expression may be used as a marker of irregular cell proliferation. However, when compared with proliferation markers, highly unrelated deviations can be seen for the reasons stated above (22–24). For these reasons, we conclude that the results of this present study are incompatible.

In the literature, P16 expression has been shown to be correlated with clinicopathological factors in breast cancer (25). In another study, p16 has been reported as the main mechanism of cell cycle deregulation in invasive breast cancer (26). In the study by Salih et al., p16 protein expression was associated with high histological grade, lymph node metastasis and poor prognosis. They suggested that p16 protein expression could be a potential prognostic marker (9). In their series, Jovanovic et al. found an increased proliferation index determined by Ki-67 nuclear expression associating increased p16 expression (27). Similarly, in this present study, p16 score was found to be associated with increased Ki-67 index. In this study, no expected association of a high p16 expression was demonstrated with poor prognosis and histopathological types, although high p16 expression was found to indicate a poorer progression of the disease.

The role of Ki-67 in the cell cycle is still unclear, despite many studies trying to establish the function of Ki-67 during the cell cycle. Ki-67 is associated with common histopathological parameters of breast cancer and there is a powerful correlation between Ki-67 expression and histological grade, since both parameters are associated with proliferation. In many studies, Ki 67 has been shown to be a significant predictor for survival and tumor recurrence in breast cancer and that the proliferative activity of those tumors determined to have Ki 67 expression may reflect the aggressivity of the disease (7, 11). Parallel to the findings in the literature in this present series, a high Ki-67 index was associated with advanced tumor stage and poor histopathological parameters and in conjunction with this finding, survival was found to decrease and a higher rate of mortality was observed in these patients.

Several limitations of this study should be acknowledged. First, the retrospective single-center design limits the generalizability of our findings and introduces the possibility of selection bias. Second, although immunohistochemical scoring was performed by two blinded pathologists with substantial interobserver agreement, the semi-quantitative 0–4 percentage-based system for PCNA and p16 has not been universally validated and lacks intensity scoring, which may have contributed to the absence of an independent prognostic signal for these two markers (12, 28). Third, Ki-67 was scored manually using the hot-spot method; while consistent with current IKWG recommendations, manual counting remains susceptible to interobserver variability and the absence of digital image analysis is a recognized limitation (12, 28). Fourth, a formal multivariate Cox proportional-hazards analysis was not performed due to the limited number of mortality events (n=20) over the follow-up period, which precluded statistically robust adjustment for the relevant covariates; this should be addressed in larger, preferably multicenter, prospective cohorts.

The absence of an independent prognostic association for PCNA and p16 in our series, despite an inter-marker correlation, deserves biological and technical comment. From a biological standpoint, PCNA participates not only in DNA replication but also in DNA damage repair, chromatin remodeling and translesion synthesis; its long half-life (>20 hours) allows persistent expression in cells that have already exited active proliferation, blunting its specificity as a proliferation marker compared with Ki-67 (11, 22, 29). p16 (CDKN2A) has a dual biological role: while loss of p16 is a hallmark of cell-cycle deregulation in tumors with intact Rb, paradoxical overexpression of p16 may occur as a compensatory response to Rb pathway inactivation, particularly in aggressive subtypes such as triple-negative breast cancer (14, 15, 26). This bimodal behavior can dilute any monotonic association between p16 expression and outcome in unselected breast cancer cohorts. From a technical standpoint, our reliance on a percentage-only semi-quantitative score, the lack of intensity scoring for p16, and the use of a single PCNA antibody clone may have further reduced the discriminative power of these markers.

Our findings are consistent with recent reports highlighting the need for standardized scoring of Ki-67 across institutions (12, 14, 28). The 2021 IKWG and St. Gallen recommendations propose a low-Ki-67 (<5%) and high-Ki-67 (≥30%) threshold for clinical decision making; the intermediate range remains a ‘gray zone’ where reproducibility is poorest and where digital image analysis may be of particular value. When viewed through the lens of molecular surrogate subtypes, our data confirm the well-established observation that Ki-67 segregates Luminal A from Luminal B and from non-luminal subtypes, and that proliferation-driven mortality is concentrated in HER2-enriched and triple-negative tumors (14, 15, 30). The absence of an independent prognostic signal for PCNA and p16 across subtypes argues against their routine adoption in clinical practice and supports current guidance that places Ki-67, when measured under standardized conditions, as the proliferation marker of choice.

## Conclusion

It was established that PCNA, p16 and Ki 67 scores were not closely associated with clinical and histopathological variables. Of these proliferation indices, only the Ki-67 index could be useful for predicting the prognosis. More evidence is required to create individualized treatment plans according to proliferation indices.

## Data Availability

The data supporting the conclusions of this study will be made available by the corresponding author upon reasonable request.

## Supplementary material

Supplementary Figure 1 Representative immunohistochemical staining of PCNA in breast carcinoma across the five-tier semi-quantitative scoring system (0–4), based on the percentage of tumor cells showing nuclear positivity. **(A)** Score 0: no staining or <1% positive nuclei. **(B)** Score 1: 1–10%. **(C)** Score 2: 11–25%. **(D)** Score 3: 26–50%. **(E)** Score 4: 51–100%. PCNA shows nuclear staining (brown, DAB chromogen; hematoxylin counterstain). Each panel is from a different patient. Original magnification ×200; scale bar = 50 µm.

Supplementary Figure 2 Representative immunohistochemical staining of p16 (clone E6H4) in breast carcinoma across the five-tier semi-quantitative scoring system (0–4). **(A)** Score 0: no staining or <1% positive tumor cells. **(B)** Score 1: 1–10%. **(C)** Score 2: 11–25%. **(D)** Score 3: 26–50%. **(E)** Score 4: 51–100%, showing diffuse block-positive nuclear and cytoplasmic staining. p16 immunoreactivity was scored as combined nuclear and cytoplasmic staining (brown, DAB chromogen; hematoxylin counterstain). Each panel is from a different patient. Original magnification ×200; scale bar = 50 µm.
