# Supplementary figures and images for: The prognostic significance of immunohistochemical expressions of proliferating cell nuclear antigen, P16 and Ki-67 in breast cancer
Source: Front Oncol. 2026 Jul 16;16:1779553. doi: 10.3389/fonc.2026.1779553 (PMC13422137; doi:10.3389/fonc.2026.1779553)

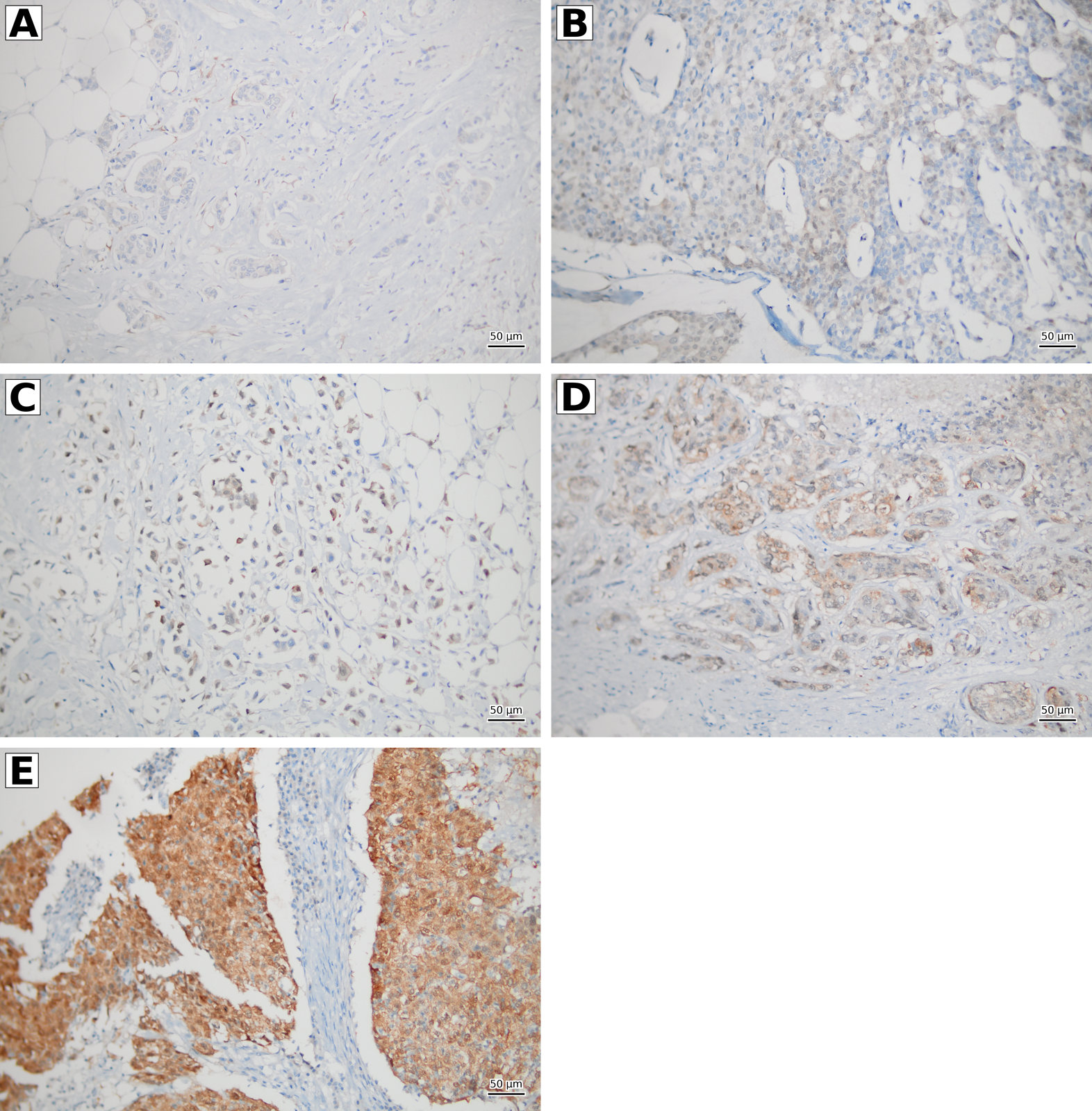

Supplement: Supplementary Figure 1 — Representative immunohistochemical staining of PCNA in breast carcinoma across the five-tier semi-quantitative scoring system (0–4), based on the percentage of tumor cells showing nuclear positivity. (A) Score 0: no staining or <1% positive nuclei. (B) Score 1: 1–10%. (C) Score 2: 11–25%. (D) Score 3: 26–50%. (E) Score 4: 51–100%. PCNA shows nuclear staining (brown, DAB chromogen; hematoxylin counterstain). Each panel is from a different patient. Original magnification ×200; scale bar = 50 µm. [file Image1.png]

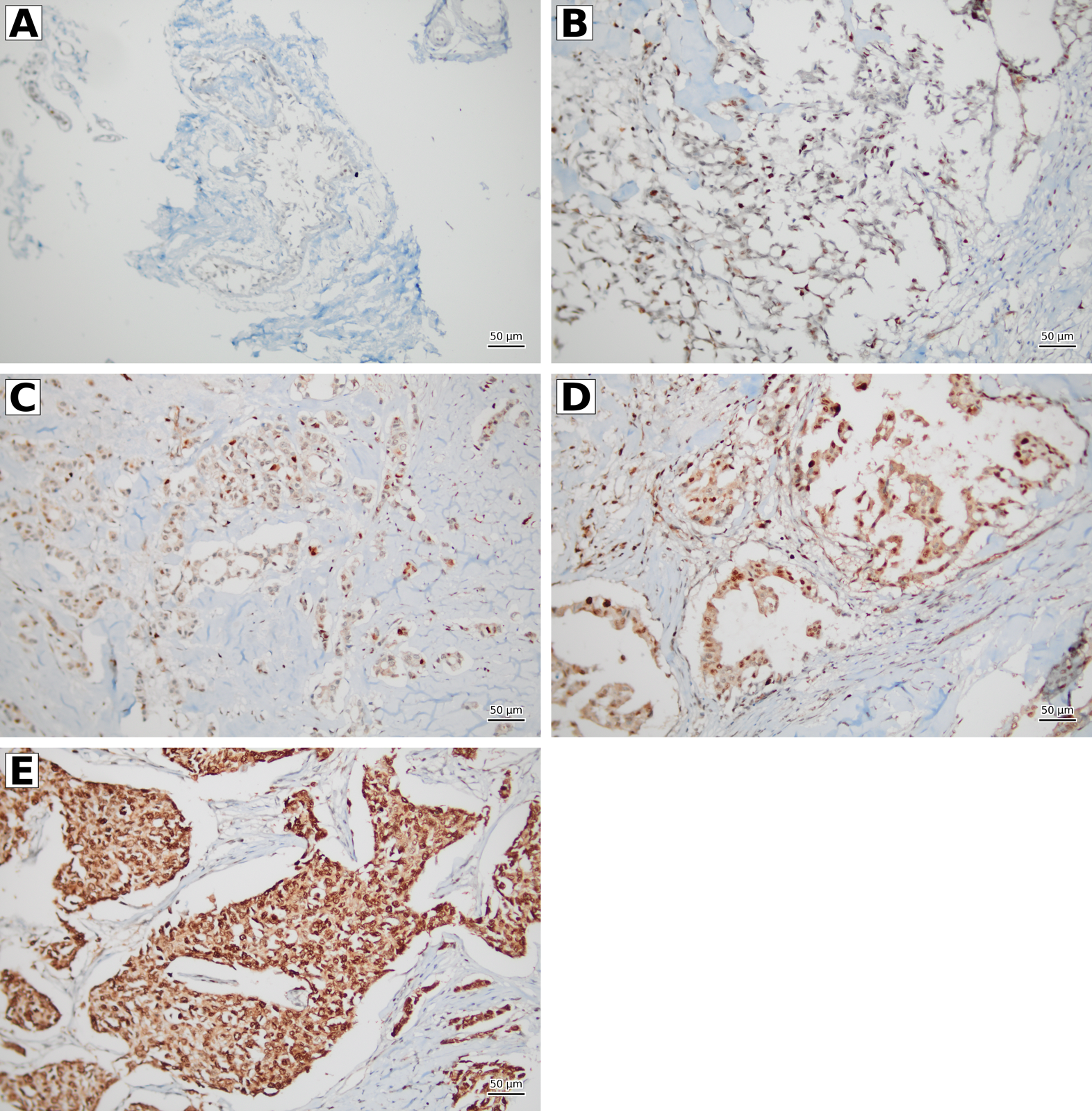

Supplement: Supplementary Figure 2 — Representative immunohistochemical staining of p16 (clone E6H4) in breast carcinoma across the five-tier semi-quantitative scoring system (0–4). (A) Score 0: no staining or <1% positive tumor cells. (B) Score 1: 1–10%. (C) Score 2: 11–25%. (D) Score 3: 26–50%. (E) Score 4: 51–100%, showing diffuse block-positive nuclear and cytoplasmic staining. p16 immunoreactivity was scored as combined nuclear and cytoplasmic staining (brown, DAB chromogen; hematoxylin counterstain). Each panel is from a different patient. Original magnification ×200; scale bar = 50 µm. [file Image2.png]
